# Rapidly Progressive Glomerulonephritis Associated With IgA Nephropathy and C3 Deposits in a Patient With Chronic Hepatitis B

**DOI:** 10.7759/cureus.70619

**Published:** 2024-10-01

**Authors:** Jennifer Wiese, Nayiri A Derian, M'hamed Turki, Tejas Joshi, Ahlim Alsanani, Anjali Satoskar

**Affiliations:** 1 Internal Medicine, Marshall University Joan C. Edwards School of Medicine, Huntington, USA; 2 Gastroenterology, Marshall University Joan C. Edwards School of Medicine, Huntington, USA; 3 Gastroenterology and Hepatology, Marshall University Joan C. Edwards School of Medicine, Huntington, USA; 4 Nephrology, Marshall University Joan C. Edwards School of Medicine, Huntington, USA; 5 Pathology, The Ohio State University College of Medicine, Columbus, USA

**Keywords:** acute kidney injury, hepatitis b, iga nephropathy, non-oliguric renal failure, rpgn

## Abstract

Hepatitis B-associated glomerulonephritis (GN) has been recognized for decades. However, only a few cases of IgA nephropathy (IgAN) in a setting of rapidly progressive glomerulonephritis (RPGN) associated with chronic hepatitis B virus (HBV) have been described. Herein, we report the case of a 42-year-old Asian female with a past medical history significant for chronic HBV on entecavir, hypertension, chronic kidney disease, and newly diagnosed breast cancer, who underwent elective bilateral mastectomy and breast augmentation. Post-operatively, she developed non-oliguric acute kidney injury and proteinuria. Renal biopsy revealed active focal crescentic and necrotizing GN with IgA and C3 deposits. Systemic autoimmune-associated and other infection-related GN were ruled out. IgAN in a setting of RPGN associated with chronic HBV was suspected.

## Introduction

Relationships between glomerulonephritis (GN) and hepatitis B virus (HBV) have been reported since 1971 [1]. However, only a few cases of IgA nephropathy (IgAN) in a setting of rapidly progressive GN (RPGN) associated with chronic hepatitis B have been described. RPGN is very rare. In the United States, the estimated incidence rate of RPGN is about seven cases per million. The incidence of HBV-related GN, on the other hand, ranges from 0.1% to 25% [2,3], manifesting as membranous nephropathy (MN), membranoproliferative glomerulonephritis (MPGN), mesangial proliferative GN, minimal change nephropathy, IgAN, and focal segmental glomerulosclerosis (FSGS) [2].

MN and MPGN represent the most common renal glomerular syndrome in HBV carriers [3]; however, IgAN is the most common glomerular disease worldwide [2]. IgAN has been considered an indolent and benign disease, with slow progression to chronic kidney disease (CKD) [4]. However, there are cases with more aggressive forms resulting in extensive crescent disease that presents as acute renal failure (AKI) [5]. Further studies are necessary to find improved standardized treatment of HBV-associated IgA in a setting of RPGN. Herein, we present a 42-year-old Asian female with HBV infection on entecavir, who developed AKI after undergoing bilateral mastectomy.

## Case presentation

**Figure 1:**
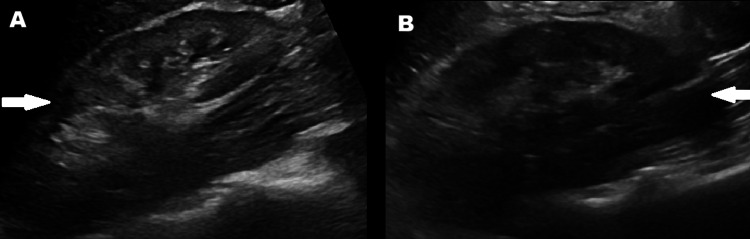
Ultrasound of the right kidney (A) and left kidney (B) showing normal ultrasound appearance (arrows).

A 42-year-old Asian female with chronic HBV, hypertension, CKD stage IV, and newly diagnosed ductal carcinoma in situ underwent elective bilateral mastectomy and breast augmentation. Preoperative laboratory values revealed mild elevated creatinine of 1.8 mg/dL (Table 1). During surgery, she developed hypotension with blood pressures ranging between 84-95 and 48-55 mmHg. Post-operatively, the patient was found to have acute-on-chronic anemia, non-oliguric AKI, and proteinuria. Renal ultrasound was non-revealing (Figure 1). Nephrology was consulted, and serological workups, including myeloperoxidase (MPO), antinuclear antibody (ANA), perinuclear anti-neutrophil cytoplasmic antibodies (pANCA), cytoplasmic anti-neutrophil cytoplasmic antibodies (cANCA), complement hemolytic activity (CH50), serum protein electrophoresis (SPEP), and urine protein electrophoresis (UPEP), were unremarkable.

**Table 1:** Laboratory results: Preoperative, postoperative, and six-weeks follow-up WBC: white blood cells; Hgb: hemoglobin; BUN: blood urea nitrogen; ALP: alkaline phosphatase; AST: aspartate aminotransferase; ALT: alanine transaminase

Tests	Preoperative Labs	Postoperative Labs	Six-Weeks Follow-Up Labs	Reference Range
WBC	7.39	15.7	12.03	(4.50-10 k/cmm)
Hgb	10.3	7.8	7.3	(12-16 g/dL)
Creatinine	1.8	3.24	4.08	(0.6-1.1 mg/dL)
BUN	35	41	56	(7-18 mg/dL)
Bicarbonate (CO2)	22	17	16	(22-29 mEq/L)
ALP	42	37	55	(45-117 U/L)
AST	13	35	16	(15-37 U/L)
ALT	17	11	22	(12-78 U/L)
Total Bilirubin	0.3	0.1	0.2	(0.2-1.0 mg/dL)

**Figure 2:**
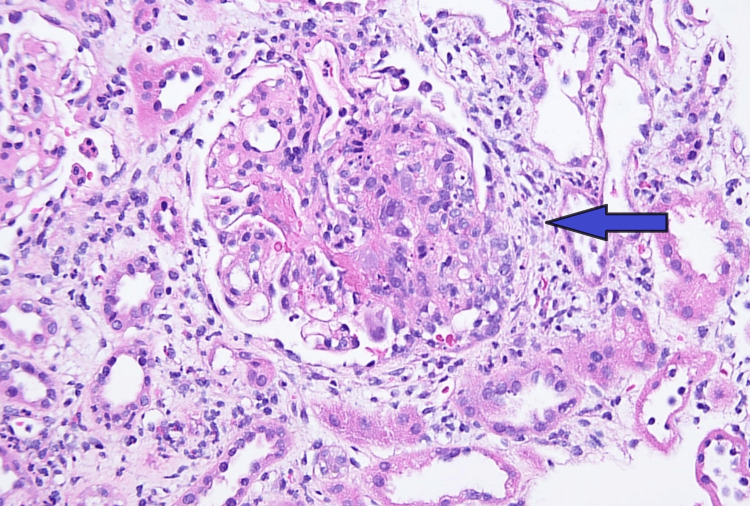
Periodic Acid-Schiff stain at 400x magnification shows focal crescentic and necrotizing glomerulonephritis (indicated by the blue arrow). There is segmental necrosis of the capillary tuft with obliteration of the capillary loops.

**Figure 3:**
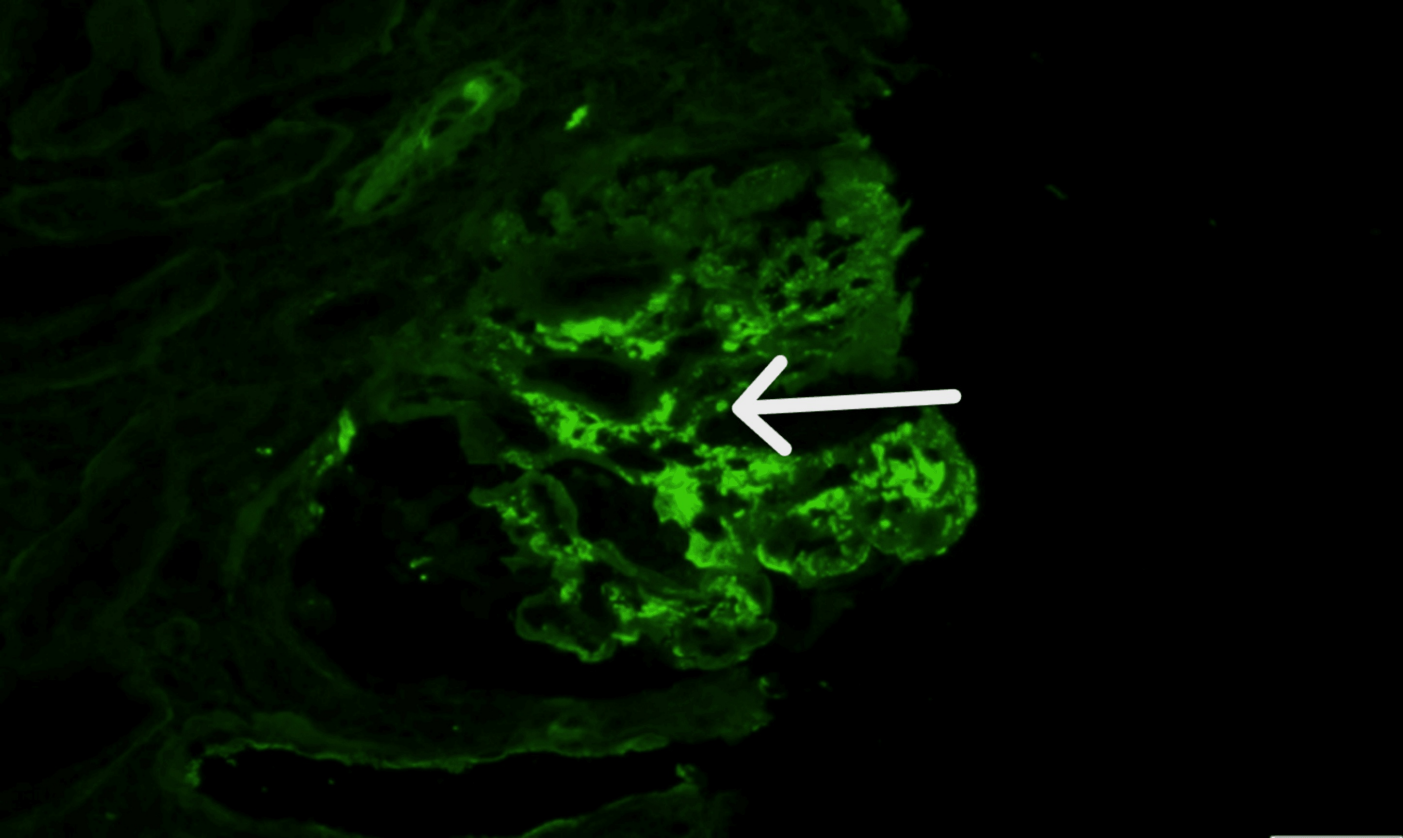
Direct immunofluorescence (DIF) staining for IgA shows 3+ granular mesangial staining in the mesangium and segmental capillary walls at 400x magnification.

**Figure 4:**
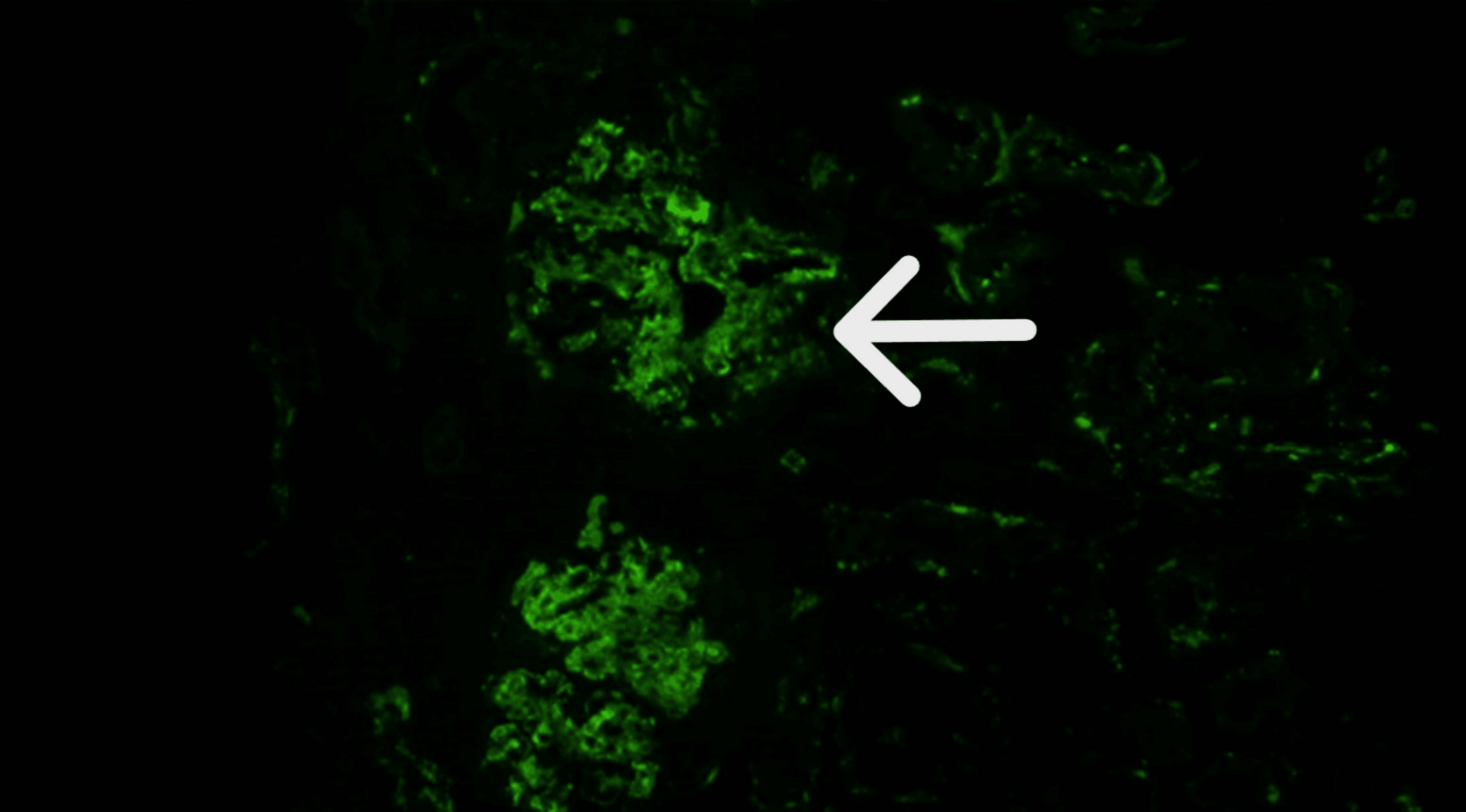
Direct immunofluorescence (DIF) staining for C3 shows 2+ diffuse granular staining in the mesangium and segmental capillary walls at 200x magnification.

The AKI was initially presumed to be secondary to acute tubular necrosis in the setting of perioperative hypotension. Fluid resuscitation was initiated, and her creatinine improved. She was stabilized and discharged with a close nephrology follow-up. Six weeks later, her creatinine increased to 4.08 mg/dL. Further work-up revealed persistent proteinuria (200 mg/dL) and active urine sediment. Renal biopsy revealed active focal crescentic and necrotizing GN (Figure 2) with IgA and C3 deposits but no antigen-antibody deposits (Figures 3-4). Immunosuppression treatment was considered early on but was delayed due to infection work-up. With the infectious disease team on board, initial workups, including blood cultures, echocardiogram, MRI, Lyme disease serology, and Nuclear Medicine white blood cell study, were negative. During the further course of evaluation, her creatinine level increased to 6.07 mg/dL, and blood urea nitrogen (BUN) rose to 97 mg/dL. Repeat serological tests, including MPO, ANA, pANCA, cANCA, CH50, SPEP, and UPEP, were negative. Serum complement levels remained within normal limits. Hepatology was consulted for possible hepatitis B-related IgAN. HBV DNA level was 390 IU/mL, and hepatitis D was undetected. Given her ethnic background and the high possibility of entecavir-resistant hepatitis B infection, entecavir was switched to tenofovir. Our nephrology team prescribed methylprednisolone 500 mg daily for three days, followed by oral prednisone 50 mg, tapered over five weeks. Atovaquone for *Pneumocystis jirovecii* pneumonia prophylaxis was also initiated. The patient’s creatinine remained stable, repeat urine creatinine ratio was 4000 mg/g, serum albumin 2.5 g, liver function tests were normal, and HBV load decreased to 40 IU/mL. The case was discussed with the American Society of Nephrology (ASN) communities and the patient was started on mycophenolate mofetil 1 g twice a day. Her creatinine stabilized at 4.77 mg/dL with an estimated glomerular filtration rate (eGFR) of 12. Several weeks later, she was hospitalized with symptomatic anemia, a urinary tract infection (UTI), and *Escherichia coli *bacteremia. She was found to have a hemoglobin (Hgb) of 4.4 g/dL and her creatinine peaked at 9.18 mg/dL. She received multiple packs of red blood cell transfusions. Unfortunately, the patient's kidney function continued to deteriorate requiring hemodialysis.

## Discussion

GN related to HBV infection most commonly occurs in endemic areas, such as some countries in Asia, Africa, and South America. In these areas, HBV infection is more likely to occur through vertical transmission. Patients born in the United States and Western Europe have a lower risk of HBV-related kidney disease due to the lower prevalence of chronic HBV infection in general [6].

The primary treatment goals for HBV infection are to prevent progression and complications of the disease such as liver cirrhosis, liver failure, and hepatocellular carcinoma (HCC) [7]. The indications for treatment are controversial and the paradigm for treatment of chronic hepatitis B is constantly evolving. The prevention of disease progression often includes the use of antiviral treatment using pegylated interferon (PEG-IFN) or nucleoside analogs, such as lamivudine, tenofovir, and entecavir. However, the combination should be considered due to increasing resistance, underscoring the necessity for further research towards novel treatments [8].

Our patient was diagnosed with HBV infection at 22 years of age and was under surveillance for many years. She initiated entecavir therapy about two years prior to presentation due to increasing alanine transaminase (ALT) value from 24 mg/dL to 66 mg/dL with hepatitis B viral load of 233,000,000 IU/mL, HBe antibody negative, HBe antigen positive, HBc antibody positive. Following treatment initiation, her viral load improved to 390 IU/mL and ALT decreased to 17 mg/dL.

HBV-related GN has been thought to be caused by a humoral immune response that leads to hepatitis B antigen-antibody (HBAg-HBAb) immune complex deposition or in-situ formation in the kidneys [1]. In addition to immune complexes, HBV DNA may also play a role in the development of IgAN via a direct cellular mechanism [6,9].

Anti-viral therapy may be considered for treating patients with HBV-related IgAN receiving immunosuppressants [10]. But, based on the current recommendations, if the patient develops RPGN with extensive crescentic disease, secondary to hepatitis B, it is recommended to give (1) antiviral medication, preferably a nucleoside/nucleotide analog; and (2) a short course of high dose steroids with or without immunosuppressants (like cyclophosphamide or rituximab) followed by a prednisone taper. There is a lack of evidence for efficacy regarding interferon, and therefore, avoided in addition to the potential to exacerbate the immune response. Antiviral therapy and HBV DNA monitoring are usually continued for 6-12 months after completing immunosuppressive therapy (depending on anti-CD20 antibody status) or until achieving therapeutic goals for hepatitis B [11].

In our patient, autoimmune disease serology as well as other possible infections, other than the patient’s underlying active hepatitis B infection, came back negative. Given her Asian ethnicity, with about 30% of patients with active hepatitis B infection showing IgAN in kidney biopsies [12], the patient was highly suspected to have hepatitis B-associated IgAN.

Due to persistent viremia, worsening kidney parameters, high suspicion of RPGN secondary to HBV-associated IgA, and high probability of resistance to entecavir, the antiviral medication was switched to tenofovir disoproxil fumarate with a renally adjusted dosage of 300 mg every three days. Her HBV viral load dropped to 40 IU/mL. The patient was also started on methylprednisolone 500 mg/d for three days then oral prednisone 50 mg, tapered over five weeks. In addition, the patient was started on mycophenolate mofetil 1 g twice a day instead of cyclophosphamide or rituximab due to possible reactivation of hepatitis B. Repeat HBV viral load increased to 80 IU/mL.

As a result of early recognition and appropriate management of HBV-associated IgA GN, hemodialysis treatment was initially delayed. Unfortunately, six weeks after the patient was started on immunosuppressive medications, she was hospitalized and was found to have sepsis secondary to *E. coli *bacteremia and severe anemia requiring multiple transfusions. Her condition was likely contributed to the adverse effects of her treatment regimen. Her kidney function continued to decline requiring subsequent hemodialysis. Repeat renal biopsy showed no active GN but scarring of the kidneys. Although newer treatment regimens have diminished the immediate mortality rate of RPGN, intermediate and long-term adverse effects are not insignificant as seen in this patient. Further studies are necessary to find improved and standardized treatment of HBV-related IgA in a setting of RPGN.

## Conclusions

This case reminds clinicians of the importance of evaluating IgAN especially in patients with chronic hepatitis B, particularly in patients with Asian ethnic backgrounds. Early detection of the etiology of the disease with appropriate initiation of treatment can consequently reduce morbidity and mortality. Treating RPGN IgAN in the setting of HBV infection requires a multidisciplinary approach and further studies are necessary to improve treatment and reduce morbidity.
